# Supplementary material for: Variance-quantitative trait loci enable systematic discovery of gene-environment interactions for cardiometabolic serum biomarkers
Source: Nat Commun. 2022 Jul 9;13:3993. doi: 10.1038/s41467-022-31625-5 (PMC9271055; doi:10.1038/s41467-022-31625-5)
Supplement: Supplementary file 3 — Description of Additional Supplementary Files [file 41467_2022_31625_MOESM3_ESM.pdf]

Description of Additional Supplementary Files

File Name: Supplementary Data 1
Description: Sample characteristics and biomarker levels by population. Note: sample sizes correspond to individuals without cardiometabolic disease or recent cancer, not pregnant, and having biomarker and genetic data available, but these numbers differ across biomarkers for vQTL and EWIS analyses due to biomarker-specific missingness and outliers. Continuous values are presented as: mean (standard deviation).

File Name: Supplementary Data 2
Description: Cardiometabolic biomarker details.

File Name: Supplementary Data 3
Description: Significant vQTLs from multi-ancestry meta-analysis across all biomarkers.

File Name: Supplementary Data 4
Description: Significant ancestry-specific vQTLs (loci not reaching significance in the meta-analysis) across all biomarkers.

File Name: Supplementary Data 5
Description: vQTL and ME locus counts and overlap.

File Name: Supplementary Data 6
Description: Replication of vQTL effects in WGHS.

File Name: Supplementary Data 7
Description: Full list of exposures as derived from PHESANT.

File Name: Supplementary Data 8
Description: Summary of exposures as derived from PHESANT.

File Name: Supplementary Data 9
Description: Significant interactions from the exposome-wide interaction study.

File Name: Supplementary Data 10
Description: Replication of BMI interactions in WGHS.

File Name: Supplementary Data 11
Description: All interaction testing results based on linear regression with anthropometric exposures impacting TG subfractions.
