# Supplementary material for: Variance-quantitative trait loci enable systematic discovery of gene-environment interactions for cardiometabolic serum biomarkers
Source: Nat Commun. 2022 Jul 9;13:3993. doi: 10.1038/s41467-022-31625-5 (PMC9271055; doi:10.1038/s41467-022-31625-5)
Supplement: Supplementary file 5 — Reporting Summary [file 41467_2022_31625_MOESM5_ESM.pdf]

Corresponding author(s): Joanne B. Cole

Last updated by author(s): May 19, 2022

Reporting Summary

Nature Portfolio wishes to improve the reproducibility of the work that we publish. This form provides structure for consistency and transparency in reporting. For further information on Nature Portfolio policies, see our Editorial Policies and the Editorial Policy Checklist.

Statistics

For all statistical analyses, confirm that the following items are present in the figure legend, table legend, main text, or Methods section.

n/a	Confirmed	
☐	☒	The exact sample size (*n*) for each experimental group/condition, given as a discrete number and unit of measurement
☐	☒	A statement on whether measurements were taken from distinct samples or whether the same sample was measured repeatedly
☐	☒	The statistical test(s) used AND whether they are one- or two-sided<br>*Only common tests should be described solely by name; describe more complex techniques in the Methods section.*
☐	☒	A description of all covariates tested
☐	☒	A description of any assumptions or corrections, such as tests of normality and adjustment for multiple comparisons
☐	☒	A full description of the statistical parameters including central tendency (e.g. means) or other basic estimates (e.g. regression coefficient) AND variation (e.g. standard deviation) or associated estimates of uncertainty (e.g. confidence intervals)
☐	☒	For null hypothesis testing, the test statistic (e.g. *F*, *t*, *r*) with confidence intervals, effect sizes, degrees of freedom and *P* value noted<br>*Give P values as exact values whenever suitable.*
☒	☐	For Bayesian analysis, information on the choice of priors and Markov chain Monte Carlo settings
☐	☒	For hierarchical and complex designs, identification of the appropriate level for tests and full reporting of outcomes
☐	☒	Estimates of effect sizes (e.g. Cohen's *d*, Pearson's *r*), indicating how they were calculated

Our web collection on statistics for biologists contains articles on many of the points above.

Software and code

Policy information about availability of computer code

Data collection

This study consisted entirely of secondary analysis of previously collected data.

Data analysis

Descriptions of many specific software programs used, along with their purpose and version, can be found in the Methods section of the manuscript. All code supporting the results described in the manuscript can be found at: https://github.com/kwesterman/vqtl-gxe.
Specific software packages and version numbers are below:
R version 3.6
R package LOLA: v1.26.0
Python: v3.6.3
Python package Scikit-learn: 0.21-0.24
LD-score regression: v1.0.1
METAL: version from 2011-03-25
OSCA: v0.45
PLINK: v1.9 and v2.0

For manuscripts utilizing custom algorithms or software that are central to the research but not yet described in published literature, software must be made available to editors and reviewers. We strongly encourage code deposition in a community repository (e.g. GitHub). See the Nature Portfolio guidelines for submitting code & software for further information.

Data

Policy information about availability of data

All manuscripts must include a data availability statement. This statement should provide the following information, where applicable:

- Accession codes, unique identifiers, or web links for publicly available datasets
- A description of any restrictions on data availability
- For clinical datasets or third party data, please ensure that the statement adheres to our policy

The UK Biobank data, including genetic and phenotypic data, can be obtained through application at https://www.ukbiobank.ac.uk/. UK Biobank will consider data applications from bona fide researchers for health-related research that is in the public interest. Genome-wide meta-analysis summary statistics for both vQTL and GEI results from this study are available for visualization and download at https://hugeamp.org/research.html?pageid=UKB-vQTL-GxE. Women's Genome Health Study data are available through collaboration; see the parent Women's Health Study website for details: https://whs.bwh.harvard.edu/. 1000 Genomes data can be accessed via FTP at: http://ftp.1000genomes.ebi.ac.uk/vol1/ftp/phase3/.

Field-specific reporting

Please select the one below that is the best fit for your research. If you are not sure, read the appropriate sections before making your selection.

☒ Life sciences ☐ Behavioural & social sciences ☐ Ecological, evolutionary & environmental sciences

For a reference copy of the document with all sections, see nature.com/documents/nr-reporting-summary-flat.pdf

Life sciences study design

All studies must disclose on these points even when the disclosure is negative.

Sample size	The sample size for the primary analysis was limited by the overall sample available from the UK Biobank cohort, which is substantial and has supported genetic discovery for many different phenotypes and analysis approaches.
Data exclusions	Individuals whose genetically-defined ancestry did not correspond to one of four pre-established groups were not included in the primary analysis, based on our original analysis plan.
Replication	The WGHS dataset was used to replicate the statistical association tests corresponding to both vQTL and GEI results derived in the primary UKB analysis. Replication was attempted for all biomarkers available in the WGHS dataset (10 of 20 biomarkers).
Randomization	Randomization was not relevant to this observational study.
Blinding	Blinding was not relevant to this observational study.

Reporting for specific materials, systems and methods

We require information from authors about some types of materials, experimental systems and methods used in many studies. Here, indicate whether each material, system or method listed is relevant to your study. If you are not sure if a list item applies to your research, read the appropriate section before selecting a response.

Materials & experimental systems

n/a	Involved in the study	
☒	☐	Antibodies
☒	☐	Eukaryotic cell lines
☒	☐	Palaeontology and archaeology
☒	☐	Animals and other organisms
☒	☐	Human research participants
☒	☐	Clinical data
☒	☐	Dual use research of concern

Methods

n/a	Involved in the study	
☒	☐	ChIP-seq
☒	☐	Flow cytometry
☒	☐	MRI-based neuroimaging
